# Morphology of sternum in patients with Scheuermann's kyphosis: is it different from that of healthy adolescents?

**DOI:** 10.1186/1748-7161-10-S1-P15

**Published:** 2015-01-19

**Authors:** Xu Sun, Zhou Wang, Zhonghui Chen, Xi Chen, Zezhang Zhu, Yong Qiu, Feng Zhu, Bangping Qian

**Affiliations:** 1 Spine Surgery, Affiliated Drum Tower Hospital of Nanjing University Medical School, Nanjing 210008, China

## Objective

To investigate the morphological characteristics of sternum in patients with Scheuermann's disease (SD) and to explore the role of sternum growth in the pathogenesis of SD kyphosis.

## Methods

20 SD patients with thoracic kyphosis aged 10 to 18 years were included in this study. 30 healthy volunteers were recruited to serve as controls. Computed tomography 3D reconstruction of the total sternum in supine position was performed. The length, width, thickness of manubrium and mesosternum was measured respectively. Meanwhile, the angle (α) between the axis of manubrium and that of mesosternum were also measured. The size of sternum and angle α were compared between SD patients and normal controls using the Studenst test.

## Results

No significant differences were found between SD patients and normal controls in terms of age and gender (P>0.05). The length, width, thickness of the manubrium was 4.7±0.8cm, 5.5±0.6cm, 0.9±0.1cm and of mesosternum was 11.4±1.1cm, 2.5±0.3cm, 1.2±0.1cm in SD patients, respectively. In normal controls, the length, width, thickness of the manubrium was 4.5±0.7cm, 5.5±0.3cm, 0.9±0.1cm and of mesosternum was 10.7±1.2cm, 2.6±0.3cm, 1.0±0.2cm, respectively. The length, thickness of mesosternum were significantly smaller in SD patients than that in healthy controls (P=0.02). The angle α was significantly larger in SD patients than that in controls (34±5° versus 28±2°, P=0.01).

## Conclusion

Probably the smaller length of sternum and abnormal articulation of manubrium and mesosternum increases the compressive forces on the vertebral endplates anteriorly, which might be involved in the pathogenesis of thoracic kyphosis of SD.

